# Non-Thermal Stabilization Strategies for Rice Bran: Mechanistic Insights, Technological Advances, and Implications for Industrial Applications

**DOI:** 10.3390/foods14091448

**Published:** 2025-04-22

**Authors:** Lu Zhou, Jiangqi Huang, Yutong Du, Fanghao Li, Wenbin Xu, Chenguang Zhou, Siyao Liu

**Affiliations:** 1 School of Food and Biological Engineering, Jiangsu University, Zhenjiang 212013, China; 2 School of Pharmacy, Jiangsu University, Zhenjiang 212013, China

**Keywords:** rice bran, lipase, rancidity, stabilization, non-thermal technologies

## Abstract

Rice bran, a major byproduct of rice processing, is rich in unsaturated fatty acids, high-quality proteins, and bioactive compounds such as γ-oryzanol and ferulic acid. However, its poor storage stability and susceptibility to hydrolytic and oxidative rancidity critically limit industrial exploitation. Recent advances in non-thermal stabilization technologies—valued for their energy efficiency, scalability, and nutrient preservation—offer promising solutions. This review systematically elucidates the enzymatic and microbial mechanisms driving bran rancidity, emphasizing lipase and lipoxygenase activity, and critically evaluates the efficacy of emerging non-thermal strategies. Key findings highlight the superiority of non-thermal methods: cold plasma reduces lipase activity by 70% within 5 min via reactive oxygen species-induced structural disruption; ultra-high pressure preserves 95% of γ-oryzanol by selectively breaking hydrogen bonds in enzymes; high-energy electron beam irradiation suppresses rancidity markers by 45–78%; and enzymatic stabilization with immobilized papain achieves 78% lipase inactivation while retaining <5% nutrient loss. Compared to thermal approaches, non-thermal technologies enhance bioactive retention, while extending shelf-life by 2–3 weeks. By addressing challenges such as microbial synergy, parameter optimization, and industrial scalability, this review provides actionable insights for deploying green, energy-efficient strategies to valorize rice bran into functional foods and nutraceuticals, aligning with global demands for sustainable ingredient innovation.

## 1. Introduction

Rice bran, accounting for 5–8% of the total mass of rice, is a key byproduct of rice milling. It is not only an important source of macronutrients, e.g., 11–17% protein, 15–20% lipids, and 20–51% dietary fiber, but also contains bioactive substances like γ-oryzanol and tocotrienol, offering substantial nutritional and economic value [1,2]. Fresh rice bran is highly susceptible to hydrolytic and oxidative rancidity, with a shelf life typically limited to only around 5 days, which severely restricts its industrial exploitation [3,4]. Additionally, lipid peroxidation byproducts accelerate protein carbonylation in rice bran, reducing digestibility and diminishing essential amino acid availability [5,6]. Furthermore, lipoxygenase (LOX)-mediated oxidation degrades heat-sensitive micronutrients, retaining <40% of carotenoids and incurring >60% vitamin E loss in untreated bran [7].

When subjected to proper stabilization treatments, rice bran becomes a valuable source of protein, essential unsaturated fatty acids, tocopherols, ferulic acid derivatives, and dietary fiber [8]. Stabilized rice bran can be used to produce instant rice bran powder, and gluten-free bread. Rice bran fiber can be incorporated into products such as cakes [9], noodles [10], and biscuits [11] to increase dietary fiber content [12]. Due to its low allergenicity, rice bran protein is also suitable for use in nutritional powders, beverages, and infant foods [13]. While traditional thermal stabilization methods, such as dry heat and microwave treatment, can suppress enzymatic activity, the high temperatures involved lead to protein denaturation and vitamin degradation, limiting their applicability [14,15,16].

In contrast, non-thermal stabilization techniques, which inactivate enzyme activity at room or low temperatures through physical or chemical means, offer a promising approach to overcoming the challenges of high-value utilization [17,18]. For instance, cold plasma can reduce lipase activity by 70% within five min by disrupting its three-dimensional structure using reactive oxygen species (ROS) [19]. Ultra-high pressure (200 MPa) destroys the hydrogen bonds of enzyme molecules while preserving thermosensitive compounds like γ-oryzanol [20]. This review focuses on the molecular mechanisms of rice bran rancidity, assesses the principles, applications, and effectiveness of non-thermal technologies, and aims to provide scientific evidence for the development of green and efficient rice bran stabilization methodologies.

### Review Methodology

This review synthesizes literature from PubMed, ScienceDirect, and Web of Science using keywords such as rice bran stabilization, non-thermal technologies, and enzyme inactivation. Inclusion criteria prioritized peer-reviewed studies focusing on mechanistic insights, scalability, and industrial applicability. Data extraction categorized methods by efficacy, nutritional retention, and technological readiness. The inclusion criteria were limited to peer-reviewed articles published in English from 2010 to the present, capturing the most relevant and recent advancements.

## 2. Structure, Nutritional Composition, and Utilization of Rice Bran

### 2.1. Morphological Structure of Rice Bran

**Figure 1:**
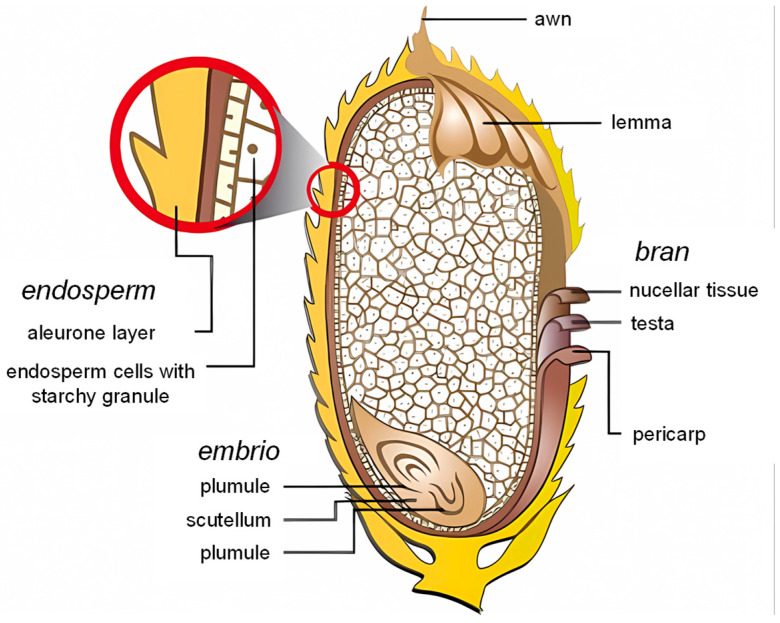
Morphological Structure of Rice grain [24].

The rice grain (*Oryza sativa* L.) is a caryopsis composed of distinct morphological layers (Figure 1). Morphologically, it consists of three principal regions: the protective outer layers, the starchy endosperm, and the embryo [21]. Externally, it is encased within an inedible husk, comprising the lemma and palea, which protect the grain during development. Upon husk removal, the edible portion consists of the fruit coat (pericarp), seed coat (testa), and nucellar tissue, collectively termed the bran. These layers are rich in lipids, proteins, bioactive phytochemicals, and hydrolytic enzymes [22]. Beneath the bran lies the starchy endosperm, constituting ~90% of the grain’s weight, which serves as the primary energy reserve. The endosperm is subdivided into the subaleurone region, containing dense protein bodies, and the central starchy endosperm, packed with compound starch granules embedded in a protein matrix. The embryo, located at the ventral base, contains embryonic axis tissues (plumule and radicle) and the scutellum, a specialized organ that interfaces with the endosperm. Grain morphology varies by cultivar, influencing size (long, medium, or short-grain), shape, and bran pigmentation (e.g., red or purple rice) [23].

### 2.2. Nutritional Composition of Rice Bran

Table 1 summarizes the content ranges for the major nutritional components in rice bran. Macronutrient analysis shows 18–23 g protein per 100 g, with dietary fiber contributing 22–32 g. Importantly, digestible protein offers a high-quality energy source, while the insoluble fiber enhances gut health by modulating microbial diversity [25]. Lipid profiling identifies linoleic acid, oleic acid, and palmitic acid as predominant fatty acids [26]. Notably, linoleic acid, an essential fatty acid, plays a critical role in regulating cholesterol metabolism, inhibiting platelet aggregation, and supporting tissue repair [27,28]. In terms of micronutrients, rice bran contains notable amounts of α-tocopherol, vitamin B_2_, and niacin. Comparative studies have shown that brown rice contains 2–2.5 times more vitamin B_1_, B_2_, and niacin than white rice, due to the complete retention of the rice bran layer [29,30].

### 2.3. Advanced Utilization Strategies of Rice Bran

As global interest in the resource utilization of cereal byproducts grows, the processing technologies for rice bran and the development of its high-value products have become key research focuses [32,33]. As an important byproduct of cereal processing, diverse applications of rice bran have extended beyond traditional feed uses, showing great promise in the food [1], pharmaceutical [34], and chemical industries [35].

#### 2.3.1. Lipid/Protein Utilization and Nutritional Advantages

In lipid applications, rice bran oil exhibits superior nutritional profiles attributed to its distinctive fatty acid composition. Refined rice bran oil typically contains 45–55% oleic acid and 30–40% linoleic acid, achieving an n-6/n-3 ratio approximating the World Health Organization-recommended 1:1 ideal balance [1]. Clinical studies have shown that this oil boasts exceptional digestibility (>96%) and offers benefits such as regulating blood lipid metabolism, improving intestinal microflora, and aiding in blood pressure reduction, making it an ideal alternative to traditional vegetable oils [36]. The protein fraction of rice bran reveals remarkable amino acid characteristics, with essential amino acids constituting 36.2% of total amino acids [37,38]. Notably, its lysine content reaches 5.1 g/100 g protein, surpassing most cereal proteins [39]. Compared to soybean protein (containing 0.1–1.5% trypsin inhibitors) and peanut protein (0.3–0.6% lectins), rice bran protein contains minimal anti-nutritional factors and low allergenicity [39]. These properties have enabled its successful incorporation into specialized nutritional products, particularly infant formulas and geriatric dietary supplements [40].

#### 2.3.2. Phenolic Compounds and Bioactive Properties

Rice bran is rich in phenolic acids, including ferulic acid, p-coumaric acid, protocatechuic acid, and vanillic acid, with ferulic acid being the most abundant [41]. Ferulic acid exists in both cis and trans isomers and is primarily present in rice bran in a bound form, typically esterified with oligosaccharides to form water-soluble feruloylated oligosaccharides [42]. These conjugates exhibit synergistic physiological properties derived from both ferulic acid and oligosaccharides, while a minor fraction occurs in the free form [42]. Studies have shown that ferulic acid can influence the migration of neutrophils to tissues and inhibit the production of inflammatory factors, exhibiting anti-inflammatory properties [43]. Ibitoye et al. [44] found that ferulic acid enhances energy metabolism, increases the generation of reactive oxygen species, and boosts the activity of the electron transport chain, while simultaneously reducing glutathione levels, thereby enhancing the antibacterial activity of quinolone antibiotics against Acinetobacter baumannii. Additionally, Chen et al. [45] developed a ferulic acid-conjugated sugar beet pulp pectin (FA-SBPP), which exhibited superior DPPH radical scavenging capacity compared to native SBPP. This enhanced antioxidant activity was attributed to structural modifications in SBPP, including altered chemical reactivity and polarity induced by feruloylation.

#### 2.3.3. Food Manufacturing Applications

Recent studies also highlight rice bran’s potential for functional applications in food processing. Incorporation of 10–15% rice bran dietary fiber enhances baked goods by increasing bread specific volume, reducing crumb hardness, and extending shelf stability [13]. At 6% supplementation level in biscuits, rice bran soluble fiber decreases in vitro starch digestibility by 30% without compromising sensory attributes [46]. In lipid-based products, research indicates that adding rice bran extract reduces the peroxide value of fish oil, extending shelf life by 2–3 times [47]. Additionally, replacing 50–75% of animal fat with rice bran oil in sausage products optimizes the n-6/n-3 fatty acid ratio with simultaneous improvements in texture [48].

## 3. Mechanisms of Rice Bran Rancidity

**Figure 2:**
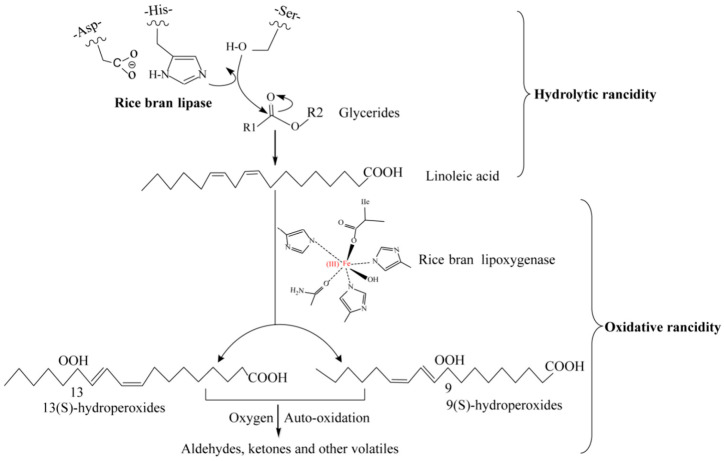
Pathways depicting the hydrolysis and oxidative rancidity of rice bran [53].

In intact brown rice, lipids are compartmentalized as lipid bodies within the aleurone layer and germ, whereas lipases are sequestered in the transverse cells of the seed coat. This spatial separation prevents lipids from undergoing rancidity [49]. However, during the milling process that produces rice bran, mechanical disruption of lipid body membranes allows lipases and oxidases in rice bran to directly interact with lipids. This interaction leads to hydrolysis and oxidation reactions, forming free radicals and volatile carbonyl compounds, which cause a rapid increase in the acid value of the rice bran [50]. Additionally, unesterified fatty acids can contribute to the development of bitter and musty flavors in the rice bran [51]. The primary rancidity pathways are summarized in Figure 2. Furthermore, the rate of rice bran rancidity is influenced by factors such as microbial activity, rice variety, and moisture content [52,53].

### 3.1. Hydrolytic Rancidity

**Figure 3:**
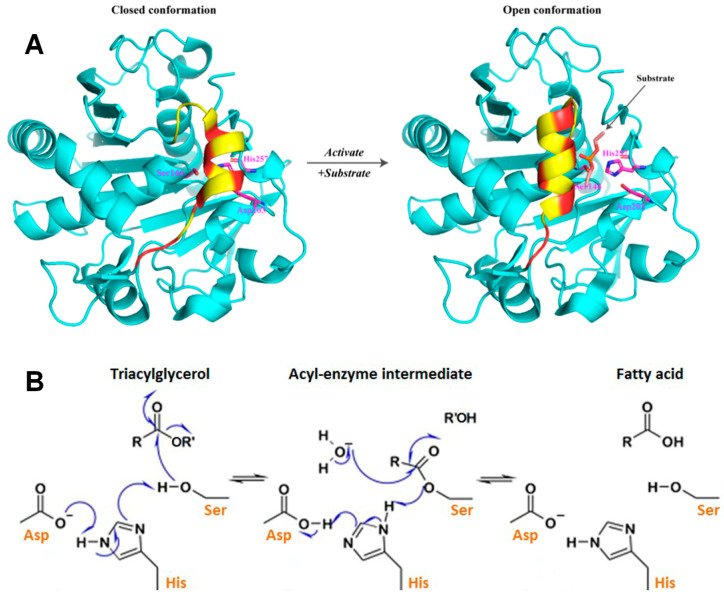
(**A**) Structural transformation of lipases from a closed conformation to an open conformation [53]; (**B**) mechanism of lipase-catalysis [58].

In rice bran, enzymatic hydrolysis of lipids is typically limited under ambient conditions. However, elevated lipase activity in the presence of optimal pH, temperature, and moisture facilitates rapid adsorption of lipases at the oil-water interface, exposing their catalytic sites for substrate binding [54]. Subsequently, the substrate enters the enzyme’s active site, where a conserved catalytic triad (Ser-His-Asp/Glu) mediates hydrolysis [53]. This triad is structurally stabilized by a pentapeptide motif within an α/β hydrolase fold scaffold (Figure 3A). Upon activation, the proton from its hydroxyl group transfers to the imidazole ring of the His residue [55,56]. Meanwhile, the carbonyl carbon of the substrate is attacked by the nucleophilic Ser residue, forming an unstable intermediate complex (Figure 3B). The His-acquired proton is subsequently transferred to the alcohol moiety, cleaving the ester bond and releasing a free alcohol. Through this reaction, the Ser residue recombines with the carbonyl bond to form an ester group, resulting in an enzyme-acyl complex [53,57]. The deprotonated His imidazole ring then abstracts a proton from a water molecule. The resulting OH^−^ ion attacks the newly formed ester bond carbonyl carbon, leading to ester bond cleavage. Finally, the His imidazole ring transfers the proton back to the Ser residue, releasing free hydrolyzed fatty acids (Figure 3B).

Generally, the catalytic efficiency of lipases depends on the spatial proximity of triglyceride ester bonds to the Ser hydroxyl group and the enzyme’s substrate-binding geometry [59]. Higher lipase activity accelerates FFA release, exacerbating lipid degradation [60]. Sinha et al. [61] further identified lipase-mediated triglyceride hydrolysis as a key contributor to off-flavor formation in rice bran lipids. Consequently, lipase inactivation strategies are widely employed to enhance rice bran stability. For instance, He et al. [62] successfully extended shelf life by suppressing lipase activity via infrared radiation.

### 3.2. Oxidative Rancidity

Lipoxygenase (LOX), a key enzyme driving oxidative rancidity in rice bran, catalyzes the peroxidation of free unsaturated fatty acids (e.g., linoleic, linolenic, and arachidonic acids) into conjugated diene hydroperoxides [3,63]. This reaction, termed lipid peroxidation, is the primary oxidative pathway in rice bran lipids. LOX exhibits its highest catalytic activity towards free fatty acids, but it can also oxidize monoglycerides and triglycerides that contain unsaturated fatty acid side chains, albeit to a lesser extent [64,65]. Typically, in the presence of molecular oxygen, LOX catalyzes the oxidation of fatty acids containing a cis,cis-1,4-pentadiene structure, generating fatty acid hydroperoxides (FAHPO) [50]. In rice bran, LOX can catalytically add an oxygen molecule to either end of dienes, such as linoleic acid and linolenic acid, producing 9-hydroperoxides (9-HPOD) and 13-hydroperoxides (13-HPOD). Among the three LOX isoenzymes identified in the rice genome, i.e., LOX-1, LOX-2, and LOX-3, unsaturated fatty acids oxidized by LOX-1 and LOX-2 primarily form 13-HPOD, whereas LOX-3 catalyzes the formation of 9-HPOD [50]. FAHPO, as unstable intermediates, degrade into small-molecule metabolites via secondary pathways—hydroxy acids through reduction, ketones via oxidation, epoxy-hydroxy derivatives via isomerization, and volatile aldehydes/acids via decomposition [66]. The accumulation of these degradation products, particularly free fatty acids and volatile aldehydes, directly elevates the acid value (AV) of rice bran, making AV a critical marker for assessing rancidity severity [31]. Furthermore, GC-MS analysis of volatile organic compounds (VOCs) has emerged as a robust method to evaluate rice bran freshness by tracking aldehyde and ketone signatures [67].

### 3.3. Other Factors

#### 3.3.1. Water Activity

Water activity (Aw) critically governs rice bran rancidity kinetics, as water molecules act as both reactants and solvents in hydrolytic rancidity [68]. Enzyme activity in foods typically correlates positively with Aw. For instance, stabilized rice bran exhibits elevated lipase activity upon moisture absorption during storage [69]. Higher Aw (>0.4) accelerates lipase activity and rancidity rates, whereas lower Aw (<0.2) slows lipid deterioration [70]. However, excessively low Aw can hinder rice bran storage: when Aw drops below the monolayer water threshold, the protective hydration layer on lipid surfaces dissipates, allowing oxygen molecules to directly contact the lipids, which accelerates oxidation [71]. Fresh rice bran typically exhibits Aw values of 0.6–0.7. Ling et al. [69] demonstrated that hot air-assisted radiofrequency stabilization optimizes storage stability at Aw 0.2–0.4, where free fatty acid accumulation and peroxide value (POV) rise minimally. Beyond Aw 0.4, FFA levels surge rapidly, while Aw <0.2 disproportionately accelerates POV elevation.

#### 3.3.2. Microbial Contamination

In addition to enzymatic activity, microorganisms also play a significant role in rice bran rancidity. Rich in lipids and proteins, rice bran serves as an ideal substrate for microbial proliferation under high humidity and temperature conditions [72]. Molds are the predominant microorganisms encountered during the growth, storage, and processing of rice [73]. Crucially, mold-derived lipases synergize with endogenous enzymes to exacerbate hydrolytic rancidity [74]. Researchers [72] isolated five lipid-hydrolyzing Bacillus strains from rice bran, revealing that these bacteria account for approximately 25% of total lipid hydrolysis, independent of lipase activity. Despite this, much of the current research continues to focus on enzymatic effects, while microbial contamination’s role in rancidity is often underexplored.

#### 3.3.3. Phenolic Compounds

Phenolic compounds, including tannins, anthocyanins, and flavonols, inhibit lipase activity and mitigate rancidity [75,76,77,78]. Zhang et al. [79] observed slower rancidity in red rice bran varieties compared to brown counterparts during accelerated aging, linking this to proanthocyanidin content. Chen et al. [80] studied different rice bran genotypes and further correlated reduced hydrolytic rancidity in purple rice bran with elevated levels of total phenolics, flavonoids, and anthocyanins, suggesting their role in suppressing lipase (LA)-mediated hydrolysis. Raghavendra et al. [81] explored how chlorogenic acid and caffeic acid inhibit rice bran lipase and found that both polyphenols interact with lipase through hydrogen bonding and hydrophobic interactions, thereby inhibiting its activity. While high phenolic content enhances rancidity resistance in colored rice bran (e.g., red and purple varieties) [82,83], the molecular mechanisms remain poorly characterized, necessitating further research into structure-activity relationships.

## 4. Non-Thermal Stabilization Technologies for Rice Bran

### 4.1. Thermal Stabilization Strategies

As delineated by rice bran rancidity mechanisms, lipase activity and water activity (Aw) constitute the primary drivers of lipid deterioration. As such, thermal stabilization protocols focus on dual objectives: (1) lipase inactivation to suppress enzymatic hydrolysis-driven rancidity, and (2) Aw reduction to impede free fatty acid accumulation, thereby extending shelf life [7] (Table 2).

Dry heating, a conventional method, employs hot air to reduce moisture and lipase activity; however, prolonged treatment compromises protein solubility and vitamin E retention [84,85,86]. Moist heat via steam or superheated steam (100–160 °C) achieves more uniform enzyme inactivation while enhancing γ-oryzanol content, though post-treatment drying is required [87,88,89]. Microwave irradiation induces rapid dielectric heating, retaining phenolic compounds and γ-oryzanol but reducing flavonoids [93,94,95]. Ohmic heating leverages electrical resistance to generate internal heat, preserving 55–92% of bioactive components while effectively suppressing lipase, yet its application is limited by sample conductivity requirements [101,102,103]. Infrared radiation combines thermal and drying effects, maintaining proximate nutrients and improving polysaccharide extraction efficiency, albeit with significant tocopherol loss [22,97,98,99,100]. Emerging radio frequency (RF) heating demonstrates superior penetration and uniformity, reducing LA/LOX activities to <19.2% and 5.5%, respectively, with minimal impact on proteins and lipids [90,91,92]. Extrusion utilizes thermo-mechanical shear to inactivate LOX (>99%) and LA (residual~32%), increasing soluble fiber but degrading heat-sensitive phytochemicals [90,91,92]. Comparatively, microwave and RF methods strike a balance between enzyme inactivation efficiency and nutrient retention, while extrusion suits large-scale stabilization despite structural damage. Optimal approaches should align with targeted applications, weighing factors such as energy consumption, scalability, and preservation of functional components like tocopherols and phenolics.

### 4.2. Non-Thermal Stabilization Technologies

In contrast to conventional thermal strategies, which often degrade heat-sensitive nutrients during enzymatic inactivation, non-thermal technologies enable enzyme suppression at ambient or low temperatures via physical (e.g., reactive species, pressure) or biological (e.g., protease catalysis) mechanisms. These approaches address hydrolytic and oxidative rancidity while maximizing nutrient retention, positioning them as transformative solutions for high-value rice bran valorization. Table 3 provides a systematic comparison of four key non-thermal methods.

#### 4.2.1. Low-Temperature Plasma

**Figure 4:**
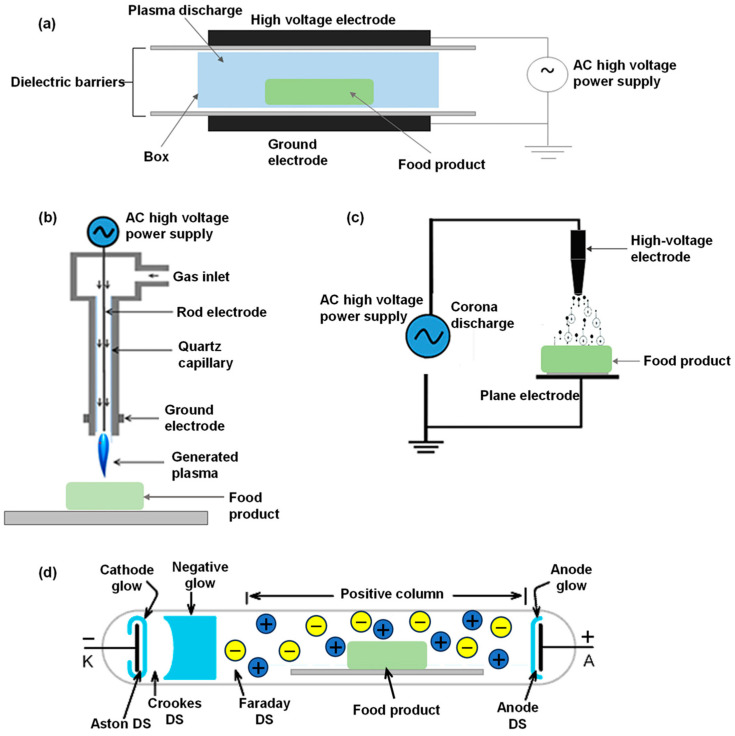
Different configurations of cold plasma designs: (**a**) dielectric barrier discharge, (**b**) plasma jet, (**c**) corona discharge, and (**d**) glow discharge [121].

Plasma is commonly referred to as the fourth state of matter, and it exhibits unique physicochemical properties. Physically, it constitutes an ionized gas comprising ions, electrons, and neutral particles, remaining electrically neutral overall [112,113]. Plasma is categorized as high- or low-temperature based on operational thermal conditions. High-temperature plasma (ion energy: 0.1–4.0 eV; current: 1–100 A) facilitates intense chaotic motion, primarily applied in mechanical machining and refractory metallurgy. In contrast, low-temperature plasma generates ions with energies between 0.03 and 0.05 eV, and its system temperature is close to room temperature [104]. This prevents any adverse effects on the treated material that may arise from extreme temperature changes. Currently, low-temperature plasma technology has found widespread applications in fields such as catalytic reactions [105,114], wastewater treatment [115], sterilization [116,117], and food processing [118,119,120]. Figure 4 illustrates the various types of low-temperature plasma generation.

The stabilization mechanism of low-temperature plasma operates via dual pathways: energy-efficient ion fluxes directed by external electric fields interact with material surfaces to induce pitting and microcracks, thereby altering physicochemical properties [122,123], while reactive species such as excited-state particles, reactive oxygen/nitrogen species (ROS/RNS), and hydroxyl radicals degrade microbial contaminants and suppress enzymatic oxidation, collectively enhancing material stability [124,125,126]. However, ROS such as ozone—generated in oxygen-containing atmospheres—pose oxidation risks to unsaturated lipids in rice bran. To address this, ozone-free plasma configurations using inert gases (e.g., nitrogen, argon) or vacuum-mode systems require precise parameter optimization to suppress ozone generation. Compared to conventional thermal methods, low-temperature plasma demonstrates superior performance in preserving nutritional quality, extending shelf life, and retaining bioactive compounds [127,128].

Recent studies have increasingly explored the application of low-temperature plasma technology in cereal stability research. Tolouie et al. [129] demonstrated reductions of 60–75% in lipase and lipoxygenase (LOX) activities in wheat germ following plasma treatment. Saberi et al. [130] observed plasma-induced modulation of soluble protein degradation kinetics in cereals. Los et al. [131] reported variability in microbial inactivation efficiency depending on plasma type and grain species. Additionally, some researchers have filed patents for the use of low-temperature plasma to stabilize rice bran, proposing a method that rapidly stabilizes rice bran’s nutritional components with plasma treatment. This approach achieves >85% lipase inactivation, suppresses free fatty acid (FFA) accumulation by 40–60%, and delays oxidation onset by 2–3 weeks while preserving over 95% of native nutrients [31]. The non-thermal nature and rapid action of low-temperature plasma position it as a promising candidate for industrial rice bran stabilization, particularly for high-value nutraceutical markets. Its ability to simultaneously inactivate lipases and suppress microbial loads [132] could streamline processing by eliminating separate sterilization steps, reducing energy costs compared to thermal methods. However, scalability hinges on resolving challenges such as gas composition optimization and continuous-flow system design for bulk processing. Modular plasma reactors, currently prototyped for cereal grains [64] and legumes [133], could be adapted to rice bran lines with minimal retrofitting. Additionally, regulatory alignment and consumer education on “cold-processed” labeling are critical for market acceptance [134].

#### 4.2.2. High-Energy Electron Beam (HEEB) Irradiation

Electron beam irradiation processing technology involves the use of γ-rays, X-rays with energies below 5 MeV, or 10 MeV electron beams to treat food through the physical, chemical, and biological effects of ionizing radiation interacting with matter. These processes are employed for disinfection, sterilization, pest control, and preservation [106,107]. Among these, γ-ray technology is the most developed, and 60Co γ-rays are commonly used for sterilizing and preserving food [135]. However, the irradiation methods have not proven effective in stabilizing rice bran. In contrast, high-energy electron beam (HEEB) irradiation—characterized by operational safety, precise controllability, scalability, and elimination of toxic residues—has emerged as a superior stabilization method for rice bran [136].

**Figure 5:**
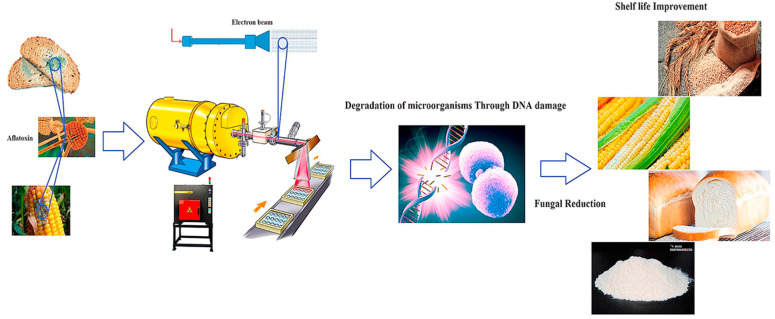
Electron beam irradiation of cereal and cereal-based products [139,142].

The principle behind stabilizing rice bran with HEEB irradiation is [137,138]: HEEBs generated by an accelerator directly damage the DNA within the active cells of rice bran or indirectly irradiate water and small molecules, generating active free radicals such as ·H and ·OH. These radicals then cross-link with nuclear materials. Low irradiation doses (2–6 kGy) effectively suppress pest activity and hydrolytic rancidity without compromising nutritional integrity. Figure 5 illustrates the configuration of HEEB equipment. The advantages of this technology include: (1) Directed electron beam radiation that maximizes resource utilization; (2) High efficiency, with treatment capacity 2.8 to 3.0 times that of γ-rays at the same power; (3) Flexible adjustments, with the ability to quickly alter the radiation dose by adjusting the conveyor speed; and (4) Energy-efficient and safe operation, with radiation easily stopped by turning off the power [139,140,141].

Shad [143] demonstrated dose-dependent lipase inactivation in rice, with activity reduced to 54.84% of baseline at 10 kGy, while sensory and nutritional profiles remained unaffected across doses (0–10 kGy). This indicates that high-energy electron beam irradiation effectively stabilizes rice bran. Furthermore, the study showed that electron beam irradiation increases the carbonyl content of lipase as the dose increases, while decreasing the total thiol group content and reducing the secondary structure and protein content of lipase. Beyond rice bran, HEEB irradiation inhibits cell wall-degrading enzymes in kiwifruit by 45–78% while preserving cellular ultrastructure [144]. HEEB irradiation’s precision and scalability offer unique advantages for industrial rice bran stabilization, particularly in regions with stringent pest-control regulations. Its ability to suppress hydrolytic rancidity without nutrient loss aligns with clean-label trends, while throughput rates enhance cost efficiency [89,106]. However, industrial adoption requires addressing dose uniformity in dense bran matrices and equipment costs for high-power accelerators [139]. Integration with existing milling infrastructure, such as conveyor-based systems with adjustable speed, could optimize dosing for bulk processing. Pilot partnerships, such as those testing HEEB-stabilized bran in gluten-free flours [89], could validate commercial viability while ensuring compliance with global food safety standards.

#### 4.2.3. Ultra-High Pressure Stabilization

Ultra-high pressure processing (UHP) is a non-thermal technique that involves treating materials in sealed vessels at 100–1000 MPa for 20–40 min under controlled temperatures (20–40 °C). This process inactivates spoilage enzymes while enhancing food quality attributes through molecular-level modifications [145]. As one of the most industrially advanced non-thermal method, UHP achieves enzyme suppression via pressure-induced cleavage or rearrangement of hydrophobic interactions and ionic bonds in proteins, alongside structural alterations to non-covalent molecular configurations [108,109,110].

Rice bran’s enzymatic deterioration, driven by lipases, proteases, and oxidases, can be effectively mitigated through UHP. The process subjects rice bran to hydrostatic pressures exceeding 100 MPa in high-strength vessels, significantly improving storage stability by disrupting protease tertiary structures [146]. Mechanistically, high pressure destabilizes salt bridges, hydrogen bonds, and hydrophobic interactions critical for enzymatic spatial conformations. The subsequent dissociation of peptide chains disrupts the α-helix/β-sheet secondary structures, exposing catalytic sites to aqueous degradation [147,148].

Studies demonstrate that UHP at 200–500 MPa significantly reduces hydrolytic and oxidative rancidity during storage. For instance, HPP at 200 MPa for 10 min minimized fat acidity and conjugated diene formation in brown rice, preserving nutritional quality while suppressing lipase activity [149]. Hydration levels during UHP further influence outcomes: treatments at ≥30% hydration reduced microbial load and modified structural properties, inducing particle aggregation and enhancing soluble dietary fiber (8.73 to 11.03 g/100 g at 15% hydration). HPP also improved emulsifying stability across hydration levels, attributed to protein structural modifications [146]. These findings underscore HPP’s dual role in microbial safety and functional enhancement, though parameter optimization (pressure, time, hydration) is critical to balance rice bran stability and techno-functional outcomes. Despite rice bran’s inherently low moisture, UHP application typically requires transient hydration to enable pressure transmission [150]. Post-treatment drying restores the powdered form while retaining UHP-induced stability improvements. For instance, spray drying post-UHP not only reduces water activity but also enhances particle uniformity and oxidative stability through controlled matrix densification [151]. Further innovations in hybrid UHP-drying systems are critical to commercial scalability, balancing metabolic inactivation with cost-efficient, industry-aligned workflows.

#### 4.2.4. Enzymatic Stabilization

The biological method for stabilizing rice bran, also known as enzyme-based stabilization, primarily involves using proteases to break down lipases [152]. The advantages of this method include the ability to target specific enzymes under mild reaction conditions, thus preserving the nutritional components of rice bran, with the added benefit of simplicity in operation [16]. Conventional free-enzyme systems, despite high specificity and low energy input, face limitations including enzyme denaturation, poor operational control, and non-recyclability [153,154]. Recent advances address these challenges through enzyme immobilization techniques such as magnetic nanoparticle fixation [155]. Immobilized enzymes are prepared by fixing free enzymes onto appropriate carriers using physical or chemical techniques. In recent years, this technology has found broad application in the food industry, including food storage, food processing, food additives, and active peptide preparation [156,157,158]. In a comparative study, papain achieved 79% lipase inactivation after 120 min, which is comparable to trypsin (80%) and chymotrypsin (86%), while bromelain and Flavourzyme paradoxically elevate lipase activity by 12–15% [152]. Enzymatically stabilized rice bran (ESRB) outperforms thermally stabilized (TSRB) and raw rice bran (RRB) in antioxidant retention, retaining 52.89% more free phenolics and 2.23-fold higher γ-oryzanol than RRB. Post-storage analyses after 60 days at 25 °C reveal ESRB’s superior stability: free fatty acid content remains at 3.92%, contrasting sharply with RRB (15.30%) and TSRB (4.67%), attributed to sustained lipase inactivation and the antimicrobial effects of liberated phenolics.

**Figure 6:**
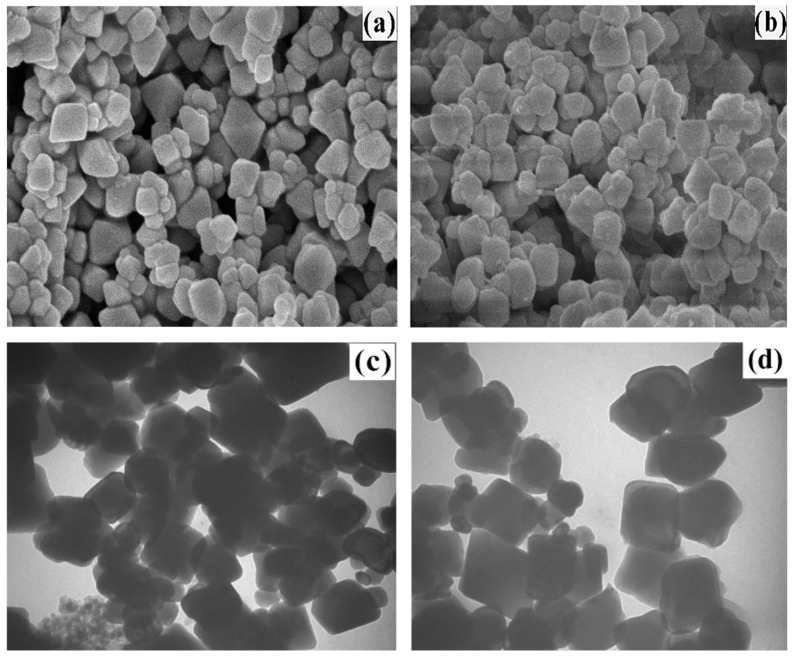
SEM and TEM images of Fe_3_O_4_/P(GMA-EDGMA-St) (**a**,**c**) and magnetic immobilized papain (**b**,**d**) [159].

Compared to free enzymes, immobilized enzymes offer advantages such as increased stability, easy recovery, reusability, and cost reduction, effectively compensating for the limitations of free enzymes [153]. A recent approach by Yu et al. [159] utilized surfactant-coated Fe_3_O_4_ nanoparticles for papain immobilization (Figure 6). Surface functionalization of these nanoparticles prevented aggregation while facilitating magnetic separation (92% recovery efficiency) and enhanced enzyme loading capacity (12.8 mg papain/g carrier). Optimal stabilization parameters (1:6 enzyme-to-substrate ratio, pH 8.0, 0.36 mg/g immobilized papain, 65 °C, 120 min) achieved sustained lipase inactivation (78% activity reduction) with minimal nutrient loss (<5% tocopherol degradation). After five cycles of reuse, the enzyme activity retention was over 88%, and after eight cycles, it was over 72%. These results demonstrate the promising potential of magnetic immobilized papain for rice bran stabilization [159].

The advantages of immobilized enzymes method include [160,161,162,163]: First, the use of nano-magnetic particles, whose porous structure increases the surface area for enzyme contact, allowing for higher enzyme concentration per unit mass of particles. Second, the rice bran suspension can continuously contact the magnetic particles and be quickly separated, improving mass transfer and heat transfer rates, which accelerates the catalytic reaction rate. Third, the reactivity of free papain is enhanced after magnetic immobilization, and it can be rapidly separated under the influence of an external magnetic field, making it convenient for repeated use. Enzymatic stabilization, particularly with immobilized systems, presents a sustainable pathway for industrial rice bran valorization. However, carrier costs (e.g., Fe_3_O_4_ nanoparticles) and batch-to-batch consistency in enzyme loading require optimization [164]. Integration into continuous bioreactors, inspired by dairy [165] and brewing industries [166,167], could enhance throughput while leveraging rice bran’s inherent phenolics for antimicrobial synergy. Regulatory frameworks must evolve to classify immobilized enzymes as processing aids rather than additives, simplifying compliance [168].

## 5. Limitations and Future Perspectives

While non-thermal stabilization methods offer significant advantages over thermal approaches, several critical limitations must be addressed to advance their industrial adoption. Microbial contamination remains underexplored but critical, as synergistic interactions between microbial lipases and endogenous enzymes exacerbate rancidity [50]. Parameter optimization across technologies—e.g., hydration levels in ultra-high-pressure (UHP) processing, irradiation doses in electron beam treatments, and enzyme-substrate ratios—requires systematic investigation to balance nutrient retention with techno-functional outcomes [169,170]. Scalability challenges persist for cold plasma and HEEB irradiation due to equipment complexity and operational costs [137,138], necessitating industrial validation [171]. At a mechanistic level, cold plasma’s molecular interactions (e.g., reactive ionic species’ roles in lipid oxidation) [172] and UHP’s pressure-time-hydration matrices demand deeper interrogation to optimize enzyme inactivation [171] while preserving structural integrity [145,173].

Future research must prioritize interdisciplinary innovations to address these gaps. Hybrid non-thermal approaches, such as combining UHP with magnetic fields, or cold plasma with enzymatic pre-treatment, could synergistically mitigate oxidative and hydrolytic rancidity. For instance, the combination of UPH treatment with magnetic field have been demonstrated to effectively mitigate the denaturation of myofibrillar proteins during the storage period of grass carp fillets [174]. Concurrently, breakthroughs in material science—such as silica-coated magnetic nanoparticles for enzyme immobilization [175] and modified biofilms with enhanced techno-functional properties [176,177] to mitigate irradiation-induced polymer degradation—are critical for optimizing scalability and sustainability [139]. These innovations not only improve process efficiency but also align with circular economy principles by enabling recyclable catalysts and bio-based packaging solutions. To fully harness these advancements, deeper mechanistic insights into enzyme-substrate interactions under non-thermal conditions are essential. While cryo-electron microscopy (cryo-EM) and synchrotron-based analyses have demonstrated success in mapping conformational dynamics of other enzymes [178], their application to lipase structural behavior under cold plasma conditions remains unexplored. Future studies could harness these techniques to resolve ROS-mediated inactivation mechanisms at near-atomic resolution, particularly by capturing transient structural deformations and oxidative damage hotspots during plasma exposure. Such insights may uncover strategies to engineer ROS-resistant lipase variants, paving the way for optimizing enzyme stability in plasma-driven bioprocessing systems [179].

## 6. Conclusions

Rice bran, a byproduct of rice milling, is rich in nutrients and bioactive compounds. However, its high lipase activity leads to rapid rancidity, limiting its shelf life and potential for high-value applications. Traditional stabilization methods, such as chemical treatments and thermal processes, have been employed to address this issue. While effective to some extent, these methods often result in nutrient degradation and are not always suitable for large-scale industrial applications. As a result, recent advancements in non-thermal stabilization technologies have gained considerable attention, offering promising alternatives to traditional methods. Technologies such as low-temperature plasma, high-energy electron beam irradiation, and ultra-high pressure processing have demonstrated significant success in improving the shelf life and nutritional quality of rice bran. These methods are particularly advantageous due to their ability to preserve bioactive compounds, prevent enzymatic degradation, and extend product stability without compromising the nutritional value of rice bran. While non-thermal stabilization technologies offer significant advantages, ongoing research is essential to optimize these methods. Addressing current limitations and exploring the synergistic effects of combining different stabilization techniques will be crucial in enhancing the efficiency, sustainability, and commercial viability of rice bran stabilization. By addressing these gaps, non-thermal technologies can propel rice bran from an underutilized byproduct to a cornerstone of sustainable, functional food innovation, aligning with global demands for nutrient-rich, eco-friendly ingredients.

## Figures and Tables

**Table 1:** Nutritional composition of rice bran [31].

Component	Average Range	Component	Average Range
Crude fat (%)	18–23	γ-oryzanol (g/kg)	0.5–5.5
Crude protein (%)	11–16		
Total dietary fiber (%)	22–32	Total Tocopherols (mg/kg)	100–150
Vitamin B1 (Thiamin) (mg/kg)	12–40	α-T (mg/kg)	50–130
Vitamin B2 (Riboflavin) (mg/kg)	1–4	β-T (mg/kg)	2–10
Vitamin B3 (Niacin) (mg/kg)	300–800	γ-T (mg/kg)	10–50
Vitamin B5 (Pantothenic acid) (mg/kg)	74	δ-T (mg/kg)	0–2
Vitamin B6 (mg/kg)	20–40		
Ca (mg/kg)	300–1200	Total Tocotrienols (mg/kg)	130–170
K (mg/kg)	5992	α-T3 (mg/kg)	38
Fe (mg/kg)	86–430	β-T3 (mg/kg)	–
Zn (mg/kg)	50–250	γ-T3 (mg/kg)	120–140
P (mg/kg)	6278	δ-T3 (mg/kg)	0–10

**Table 2:** Research on Thermal Stabilization Methods for Rice Bran.

Method	Principle	Advantages	Limitations	Ref
Dry heat	direct heating at ~120 °C to inactivate enzymes	simple and practical for commercial use; cost-effective and easy to scale	uneven heating; temperatures <100 °C ineffective; excessive heat degrades nutrients.	[84,85,86]
Moist heat	indirect heating using high-pressure/ambient steam	better preservation of nutrients compared to dry heat; more uniform heat distribution	requires precise moisture control; higher energy consumption	[87,88,89]
Extrusion	mechanical extrusion with steam injection induces structural changes and enzyme denaturation	rapid and continuous processing; stabilizes bran while improving texture	high energy consumption; expensive machinery	[90,91,92]
Microwave	dielectric heating via microwave absorption generates bulk heating, and synchronizing heat	fast and energy-efficient; minimal nutrient loss;	requires precise control of temperature to avoid over-heating; high initial equipment cost	[93,94,95,96]
Infrared	short-wavelength radiation minimizes thermal inertia and damage while suppressing enzymes activities	fast and energy-efficient; uniform heat distribution	limited material penetration depth; potential uneven heating in bulk processing	[22,97,98,99,100]
Ohmic Heating	Joule heating via electrical resistance converts current to thermal energy	rapid and uniform heating; minimal nutrient loss.	high equipment complexity; limited scalability for industrial use	[101,102,103]

**Table 3:** Non-Thermal Stabilization Methods for Rice Bran.

Method	Principle	Advantages	Limitations	Ref
Low-temperature Plasma	Reactive species (ROS/RNS) disrupt enzyme structure	Rapid, nutrient retention	Limited penetration depth	[104,105]
HEEB Irradiation	Ionizing radiation generates free radicals to crosslink DNA/enzymes	Scalable, no chemical residues	Polymer degradation at high doses	[106,107]
Ultra-High Pressure	Hydrostatic pressure disrupts hydrophobic/ionic bonds	Preserves thermosensi-tive compounds	High equipment cost	[108,109,110]
Enzymatic Stabilization	Proteolytic degradation of enzymes using im-mobilized enzymes	Targeted action, recyclable catalysts	Requires optimization of E:S ratios	[16,111]
